# Measurement of Patient-Derived Glioblastoma Cell Response to Temozolomide Using Fluorescence Lifetime Imaging of NAD(P)H

**DOI:** 10.3390/ph16060796

**Published:** 2023-05-26

**Authors:** Diana V. Yuzhakova, Daria A. Sachkova, Marina V. Shirmanova, Artem M. Mozherov, Anna V. Izosimova, Anna S. Zolotova, Konstantin S. Yashin

**Affiliations:** 1 Institute of Experimental Oncology and Biomedical Technologies, Privolzhsky Research Medical University, 10/1 Minin and Pozharsky Sq., 603005 Nizhny Novgorod, Russia; yuzhakova-diana@mail.ru (D.V.Y.); sachkova.collins@gmail.com (D.A.S.); artemmozherov@gmail.com (A.M.M.);; 2 Institute of Biology and Biomedicine, Lobachevsky State University of Nizhny Novgorod, 23 Gagarin Ave., 603950 Nizhny Novgorod, Russia; 3 Department of Neurosurgery, Privolzhsky Research Medical University, 10/1 Minin and Pozharsky Sq., 603005 Nizhny Novgorod, Russia; zolotova_anna.1997@mail.ru (A.S.Z.); jashinmed@gmail.com (K.S.Y.)

**Keywords:** glioblastoma, personalized therapy, patient-derived cell culture, fluorescence lifetime imaging FLIM, metabolism, NAD(P)H, chemotherapy, temozolomide TMZ

## Abstract

Personalized strategies in glioblastoma treatment are highly necessary. One of the possible approaches is drug screening using patient-derived tumor cells. However, this requires reliable methods for assessment of the response of tumor cells to treatment. Fluorescence lifetime imaging microscopy (FLIM) is a promising instrument to detect early cellular response to chemotherapy using the autofluorescence of metabolic cofactors. Here, we explored FLIM of NAD(P)H to evaluate the sensitivity of patient-derived glioma cells to temozolomide (TMZ) in vitro. Our results demonstrate that the more-responsive cell cultures displayed the longest mean fluorescence lifetime τm after TMZ treatment due to an increase in the protein-bound NAD(P)H fraction α_2_ associated with a shift to oxidative phosphorylation. The cell cultures that responded poorly to TMZ had generally shorter τm, i.e., were more glycolytic, and showed no or insignificant changes after treatment. The FLIM data correlate well with standard measurements of cellular drug response—cell viability and proliferation index and clinical response in patients. Therefore, FLIM of NAD(P)H provides a highly sensitive, label-free assay of treatment response directly on patient-derived glioblastoma cells and can become an innovative platform for individual drug screening for patients.

## 1. Introduction

Glioblastoma is the most aggressive form of brain tumor that accounts for ~50% of all primary malignant brain tumors. Its median survival has remained mostly unchanged for 30 years and the treatment regimen extends it up to 15 months after the initial diagnosis [1,2]. Despite several clinical trials performed in the last 15 years, the therapeutic options for primary glioblastoma remain limited. The standard-of-care therapy consists of maximal surgical resection of the aberrant tissue in combination with chemotherapy, based on the alkylating agent TMZ, and radiation treatment [2,3]. In spite of these aggressive treatments, recurrence is almost inevitable. The possible approaches for recurrent glioblastoma include re-resection, reirradiation, treatment with the anti-angiogenesis agent bevacizumab, and experimental therapies in the context of clinical trials [1,2,3]. Unfortunately, none of these approaches significantly increase survival rate. For recurrent glioblastoma patients, the six-month progression-free survival is ~15% and the overall survival is less than six months [4,5]. Two key factors pose a significant challenge in the treatment of glioblastoma—the impossibility of complete, radical resection due to highly invasive growth and the low efficacy of the treatment regimens used. The average response rate of central nervous system tumors to cytotoxic chemotherapy is the lowest among all types of cancer (9.2%) [6]. Moreover, there is extensive intra- and intertumoral heterogeneity in the variable responses of different patients to the same therapy [7,8]. It is obvious that personalized strategies for glioblastoma treatment are highly necessary.

Recently, all gliomas were classified into two groups based on the presence of mutations in the genes encoding for the enzymes isocitrate dehydrogenase 1 and 2 (IDH1/2), which play important roles in cellular metabolism, epigenetic regulation, redox states, and DNA repair [9]. IDH enzymes catalyze the conversion of isocitrate to alpha-ketoglutarate (α-KG), an intermediate in the citric acid cycle. The metabolic consequences derived from IDH mutations lead to selective vulnerabilities within tumor cells, making them more sensitive to standard and new therapeutic approaches [9,10,11]. IDH status is considered to be a valuable prognostic parameter. IDH mutations are recognized in >80% of glioma grade 2–3 cases and secondary glioblastoma (grade 4). The presence of IDH mutations is a favorable survival factor in glioblastoma (IDH-wildtype: 15 months; IDH-mutant: 31 months) [9]. However, IDH-mutated glioma of grade 2–3 may undergo malignant transformation over time and is more likely to develop a hypermutation phenotype, which is associated with worsened prognosis [10,11].

The presence of the specific molecular profile of glioblastoma highlights the need for developing effective personalized treatments, with the molecular make-up of the tumor informing the treatment regime for each individual patient [7]. Currently, two main directions in the development of personalized therapy can be distinguished: (1) selection of therapy based on full genome or exome sequencing of the tumor; (2) selection of therapy based on testing of drugs in in vitro models [12,13,14]. Despite the high informativeness of the first approach based on sequencing, it is time-consuming, expensive, and requires tissue and cellular dissociation. Another possible approach to personalized therapy is drug screening of patient-derived cells and the selection of the most effective drug(s) or regimen for individual patients based on the assessment of cellular responses. Since there are no standards for assessing therapeutic response in cellular systems, different assays are used that mainly rely on analyses of cell viability and the inhibition of proliferation using specific dyes or reporters.

One of the most promising methods for the assessment of cellular response to drug therapy is fluorescence lifetime imaging microscopy (FLIM) of autofluorescent metabolic cofactors. Unlike conventional fluorescence microscopy that captures the intensity of the emission, FLIM measures fluorescence lifetime, the time a fluorophore remains in an excited state before emitting a photon. Fluorescence lifetime is an intrinsic characteristic of a fluorophore defined by its chemical structure and molecular environment, thus existing independently of fluorophore concentration and microscope configuration. Therefore, use of FLIM is capable of monitoring conformational or microenvironmental changes in a quantitative manner with high sensitivity and molecular specificity. Being installed in a laser scanning microscope, use of FLIM provides nondestructive imaging with (sub)cellular resolution. FLIM has been extensively used for label-free analysis of the metabolic status of living cells and tissues based on the registration of fluorescence lifetime parameters of metabolic coenzymes such as reduced NAD(P)H and oxidized flavin adenine dinucleotide FAD [15,16]. These cofactors act as electron carriers in a number of biochemical reactions, primarily in energy metabolism. The forms of the cofactors with long fluorescence decay times are typically associated with mitochondrial respiratory complexes, and their contribution increases when the balance in energy-producing pathways is shifted to oxidative phosphorylation. Perturbations in energetic pathways caused by chemotherapy can result in changes in the autofluorescence intensity and/or lifetime parameters of the metabolic coenzymes, which can be observed using FLIM [17,18,19,20]. Compared to the conventional assays of treatment response, FLIM offers several advantages, such as capability to monitor dynamic changes at a single-cell level and identification of cellular heterogeneity, including the presence of non-responsive subclones with distinct metabolic features [15].

In a recent study by Morelli et al., the possibility of following the response of patient-derived glioblastoma cells to TMZ using metabolic FLIM and a live organoids model were demonstrated [21]. The authors detected a higher fraction of protein-bound NAD(P)H associated with oxidative metabolism in TMZ-treated responsive cultures compared to non-responsive ones, which was well reflected at the molecular level. However, overall, the potential of FLIM as a response assay in drug screening tests for glioblastoma has been investigated poorly.

Here, we explored FLIM of NAD(P)H to assess the sensitivity of patient-derived glioma cells to TMZ. The data from FLIM were correlated with the standard cell response assays (viability, proliferation) and the clinical statuses of patients.

## 2. Results

### 2.1. Characterization of the Patient-Derived Glioma Cell Cultures

**Figure 1:**
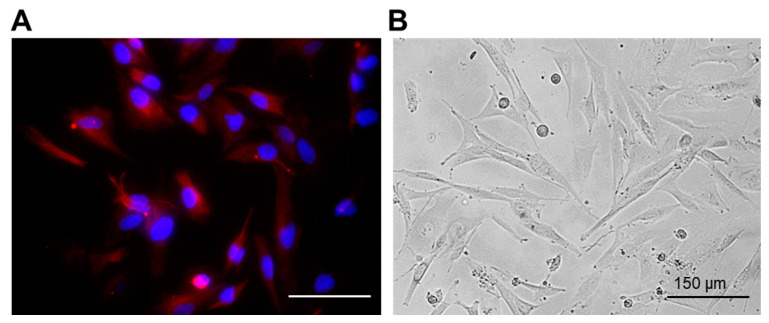
Representative immunofluorescent staining (**A**) and phase contrast (**B**) images of the patient-derived glioma cell culture (P6 sample). Red channel corresponds to GFAP expression. Cell nuclei were counterstained with DAPI (blue channel). Bar is 150 µM.

The obtained patient-derived glioma cell cultures were characterized by diffuse cell distribution and moderate or pronounced cell polymorphism (Figure 1B). All cell cultures contained numerous large cells with a fusiform morphology (fibroblast-like cells) and some amount of triangular, oval, round or irregularly shaped cells. The cells in all glioma cultures demonstrated numerous extensive cytoplasmic prolongations and pronounced cytoplasmic granularity.

To confirm the glial origin of the cells, the expression of glial fibrillary acidic protein (GFAP), the most widely used astroglial marker, was examined (Figure 1A). The expression of GFAP was observed in all obtained glioma cultures.

The proliferation index estimated with Ki67 staining varied from ~40 to 70% (Table 1).

For each cell culture, the IC50 of TMZ was determined via MTT-assay. It was found that cell cultures from different patients differed in their sensitivity to the drug. The IC50 varied over a wide range, from 476 µM to 1757 µM. On the basis of the IC50 values, a TMZ concentration of 1500 µM was selected for treatment of patient-derived glioma cultures in the FLIM response assay.

### 2.2. Cell Viability and Proliferation Index after TMZ Treatment

**Figure 2:**
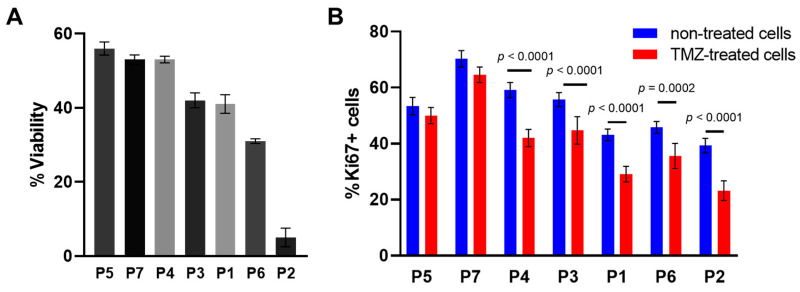
Effects of TMZ on viability and proliferation of individual glioma cell cultures. (**A**) The cell viability assay of TMZ-treated patient-derived glioma cell cultures. The MTT assay at TMZ concentration of 1500 μM, 72 h exposure. (**B**) Expression of the proliferation marker Ki67 in patient-derived glioma cells. The labels below the horizontal axis indicate the patient-derived glioma cell cultures. The data are presented as the mean ± SEM.

We assessed the cell viability and the proliferation of the patient-derived glioma cultures after TMZ treatment at 1500 µM. It should be noted that the level of cell viability varied widely in different cultures, from 56 ± 2.1% (in P7) to 5 ± 3.2% (in P2) (Figure 2A). To verify the effects of the TMZ treatment on proliferation of patient-derived glioma cell cultures, the expression of Ki67 was assessed using intracellular staining (Figure 2B). Similarly to the cell viability, the proliferative changes varied among different patient-derived glioma cultures. The most TMZ-resistant cultures, P5 and P7, demonstrated a high proliferative index and no statistically significant change under TMZ-treatment (53 ± 3.1% and 70.3 ± 2.9% in non-treated cells versus 50 ± 3.1% and 64 ± 2.8% in P5 and P7, respectively, *p* > 0.05). All other cultures showed a statistically significant decrease in the proliferation index after TMZ treatment (*p* ≤ 0.0002), with the lowest index in the most-sensitive cultures P6 and P2 (45 ± 2.1% and 39 ± 2.6% in non-treated cells versus 36 ± 4.5% and 23 ± 3.5%, *p* ≤ 0.0002).

### 2.3. FLIM of the Patient-Derived Glioma Cell Cultures after TMZ Treatment

The autofluorescence lifetime parameters of NAD(P)H were analyzed in the glioma cell cultures after TMZ treatment for 72 h and compared with those of the untreated controls. In the preliminary experiments, it was shown that shorter incubation time with TMZ did not result in pronounced metabolic changes (Supplementary Figure S3).

In untreated cells, the short lifetime value τ_1_ was 0.3 ns on average, long lifetime τ_2_ varied from 1.9 to 2.3 ns and its relative contribution α_2_ varied from 22 to 25%, and the mean lifetime τ_m_ was 0.6–0.8 ns. Pronounced differences between individual cultures suggest a high degree of intertumor metabolic heterogeneity (Supplementary Table S1, Figure S3). As expected, the initial metabolic state correlated well with the proliferation index of the cells. A good negative correlation (R^2^ = 0.75) was found between NAD(P)H τ_m_ and the Ki67 proliferative index (Supplementary Figures S1 and S2). The level of cellular metabolic variability for individual cell cultures was also different and did not change after the treatment.

**Figure 3:**
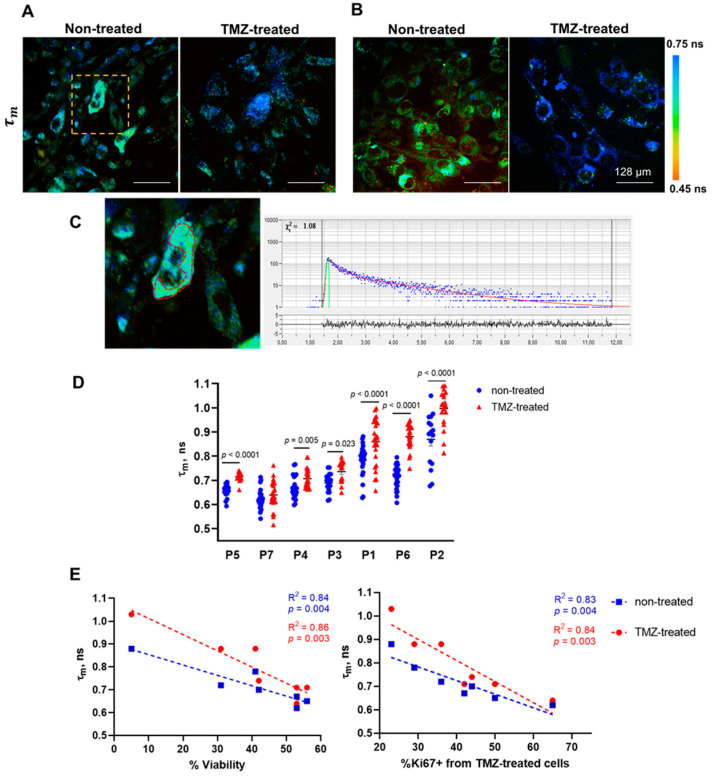
FLIM of NAD(P)H in untreated and TMZ-treated patient-derived glioma cultures. (**A**,**B**) Representative FLIM images of the mean fluorescence lifetime τ_m_ in responsive (P7) and non-responsive (P1) samples. (**C**) Enlarged area of the FLIM image (**A**) is shown by the yellow-dashed square; the red-dashed line highlights the cytoplasm zone of the cell. Typical fluorescence decay curve of NAD(P)H obtained from SPCImage software: the experimental data (blue dots), bi-exponential fit (red curve), instrument response function (green curve). (**D**) Quantification of τ_m_ in patient-derived glioma cultures without treatment and treated with TMZ (1500 µM). Scatter dot plot displays the measurements for individual cells (dots) and the mean and SEM (horizontal lines). The labels below the horizontal axis indicate the individual patients. (**E**) The Pearson correlation between NAD(P)H τ_m_ in untreated and treated cell cultures and post-treatment viability and proliferation. Scatter diagrams display the mean values for each glioma culture (dots) and the trend line.

The treatment of cells with TMZ caused changes in the fluorescence decay parameters τ_2_, α_2_ and τ_m_ in different degrees in different cell cultures. “Responsive” cell cultures showed an increase in either the protein-bound NAD(P)H fraction α_2_ associated with a shift to oxidative phosphorylation or the long lifetime component τ_2_ associated with the modified profile of NAD(P)H-binding proteins, or both, which resulted in the increase of the mean lifetime τ_m_. Of these parameters, tm demonstrated the highest linear correlation with the percentage of viable cells (R^2^ = 0.86, *p* = 0.003) and the Ki67 proliferative index (R^2^ = 0.84, *p* = 0.003) after treatment, and therefore can be considered a reliable indicator of cell response to TMZ (Figure 3C). Among the cell cultures, only P7 showed no statistical difference in τm after treatment, which correlated with a high percentage of viable cells (53 ± 1%) and a high proliferation index (64 ± 2.8%). The cultures P1, P6, and P2 demonstrated the largest difference from controls (*p* < 0.0001) in τm value and the lowest viability and proliferation rate (<35%). Concerning α_2_ and τ_2_ values, they displayed only moderate correlation with the percentage of viable cells and the proliferative index (Supplementary Figure S1). It should be noted that the resistance or sensitivity of cell cultures to TMZ treatment was preserved at a larger drug dose (2500 μM) (Supplementary Figure S4).

Of note, there was an association between the response of cells to TMZ and the initial metabolic state assessed using FLIM. Strong correlations were found for initial τm value, post-treatment cell viability (R^2^ = 0.84, *p* = 0.004), and proliferation index (R^2^ = 0.83, *p* = 0.004). The more-responsive cell cultures displayed the longest τm, which indicates their more oxidative metabolism, while the cells that did not or poorly responded to TMZ had generally shorter τ_m_ values, i.e., were more glycolytic.

Therefore, these results demonstrate that the NAD(P)H mean fluorescence lifetime is sensitive to the response of patient-derived glioma cell cultures to TMZ treatment.

### 2.4. Comparison of NAD(P)H FLIM Parameters in Cells from Primary and Recurrent Gliomas

Most of the glioma cultures in our study were obtained from IDHm patients (P1–5 and P7 cultures). Interestingly, despite the same IDH status, these cultures demonstrated a different response to the TMZ treatment. We noticed that the cultures obtained from a recurrent tumor (P1, P2) receiving the therapy responded to TMZ better than those from the primary tumors that had no previous treatment (P5, P7). 

**Figure 4:**
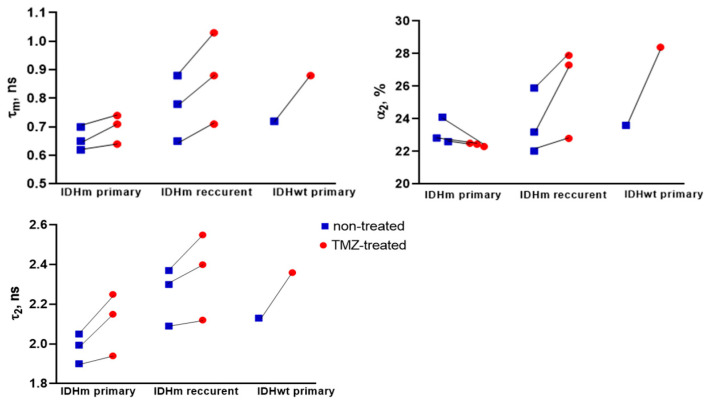
FLIM of NAD(P)H in patient-derived glioma cultures obtained from IDHm primary tumors, IDHm recurrent tumors, and IDHwt primary tumor. Quantification of fluorescence lifetime parameters τ_m_, α_2_, and τ_2_. Scatter dot plot displays the mean for individual cell cultures (dots) without treatment and treated with TMZ (1500 µM), connecting by line.

We compared the NAD(P)H FLIM parameters for IDHm primary tumors (P5, P7 and P3), IDHm recurrent tumors (P1, P2, P4), and an IDHwt primary tumor (P6) (Figure 4). The initial values of τ_2_ were statistically lower in the IDHm tumors in comparison with the IDHm recurrent tumors (*p* = 0.0455), which could be due to the change in the profile of NAD(P)H-binding proteins in the course of patient treatment.

For the cell cultures from patients with IDHm recurrent glioma, the statistically significant increase in τm after TMZ treatment was due to increases in both α_2_ and τ_2_. In contrast, in the case of IDHm primary tumors, the slight increase in τm was associated only with an increase in τ_2_. These results suggest that the previous treatment of patients may affect the metabolic status of tumor cells and further in vitro response to drug treatment.

### 2.5. NAD(P)H FLIM of Patient-Derived Cell Culture and Response to Treatment in Patients

**Figure 5:**
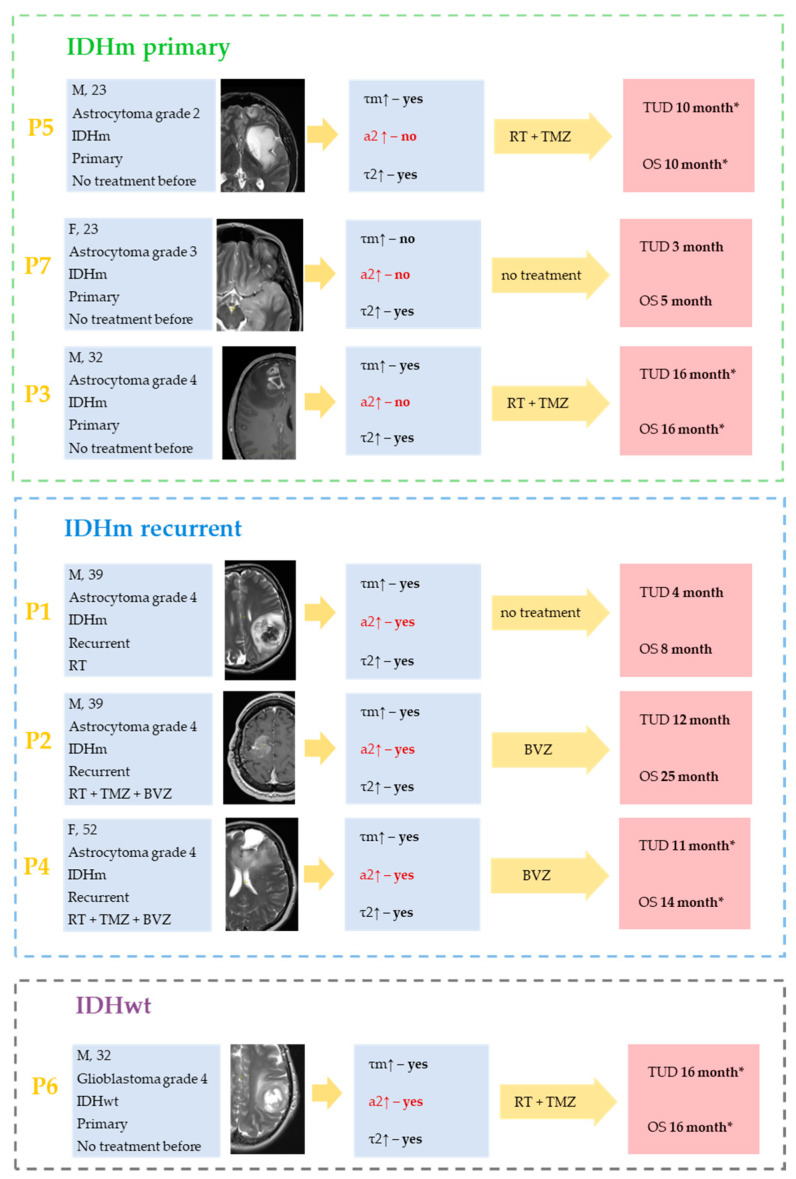
The summarized clinicopathological characteristics of patients and FLIM data for the corresponding patient-derived glioma cultures from IDHm and IDHwt glioma patients. No α_2_ increase (marked by red) may be pathognomonic for cell cultures from patients with primary IDHm glioma. For the cell cultures from patients with IDHm recurrent and IDHwt tumors, the statistically significant increase in τm after TMZ treatment was due to increases in both α_2_ and τ_2_. The absence of statistically significant increase in τ_2_ may be associated with poor prognosis in patients (P7). Yellow arrows represent the therapy obtained by the patient after the resection (RT—radiation therapy, TMZ—temozolamide, BVZ—bevazisumab). Survival rates are indicated as overall survival (OS) and time until death (TUD) from the start date of this study. Most patients are in the process of treatment and are indicated with *.

Evaluation of the relationship between patient response to treatment and the dynamics of cell culture fluorescence lifetime after TMZ therapy is difficult due to the fact that some patients are in the process of treatment. If we take the absence of tumor progression for 10 months or more as a good response, then this time was not reached in only in two patients (Figure 5, P1, P7). It should be mentioned that these two patients did not receive any treatment since the time the study started (Figure 5, P1, P7). They had a negative outcome within 3–4 months due to severe neurological deficits and the general condition of the patients. However, the dynamics of FLIM NAD(P)H lifetimes in cell cultures from both patients was different—a response to the TMZ treatment was detected in P1, but not in P7.

All cell cultures from responders, regardless of therapy (temozolamide—TMZ or bevacizumab—BVZ), showed an increase in τ_m_ after TMZ therapy (Figure 5). In no case was there a dissociation between the patient’s response to treatment and the lack of FLIM NAD(P)H lifetime dynamics in the cell culture. In P7, the absence of τ_m_ increase in the P7 patient’s cell culture was associated with poor clinical outcome.

## 3. Discussion

Metabolic reprogramming is an important biological feature of malignant tumors that affects the efficacy of the therapy. It is known that the metabolic profile of cells and the level of intratumoral heterogeneity can determine sensitivity to drug therapy, while the metabolic changes developed under treatment can modify therapeutic response [22,23]. Here, we applied metabolic FLIM to evaluate in vitro the response of patient-derived glioma cells to TMZ in order to predict the efficacy of therapy in patients.

FLIM microscopy based on endogenous fluorescence of metabolic cofactors is a promising instrument for detecting early cellular response to chemotherapy. The potential of FLIM for monitoring of tumor cell response to therapy has been demonstrated in monolayer cell cultures, tumor spheroids, and tumor xenografts in mice [17,18,19,20]. These studies with chemotherapeutic drugs having different mechanisms of action suggest that tumor response to chemotherapy is accompanied by a shift to a more oxidative metabolism, manifested as an increase in the contribution of protein-bound NAD(P)H α_2_ and the NAD(P)H mean lifetime tm. The switch to OXPHOS in cancer cells is typically associated with reduced proliferative activity [22,23].

Owing to a high sensitivity of metabolic FLIM to drug response and cellular-level heterogeneity, assessment of the treatment response of patient-derived cells in vitro in predictive drug screens for patients has been proposed. The most extensive work in this direction has been conducted by Melissa Skala’s group. Using different cancer types including breast, colon, pancreatic, head, neck, and some others, they established that optical metabolic imaging captures early metabolic changes at the single-cell level that predict later clinical response [19,20,24,25].

However, the studies on glioma models using metabolic FLIM are still limited. There is only one paper by Morelli et al. demonstrating the promise of using FLIM of NAD(P)H to evaluate drug susceptibility of patient-derived glioblastoma organoids based on an explant culture [21]. Despite the fact that organoids better resemble original tissue in terms of heterogeneity, architecture, and microenvironment than cell monolayers, it is difficult to achieve high reproducibility on this tumor model [26]. A feature of our study is the use of monolayer cultures, which allowed us to analyze NAD(P)H fluorescence lifetimes in cell cytoplasm and to identify the cellular metabolic heterogeneity more accurately. Furthermore, the passaged primary cell cultures provided a greater number of experiments with each sample and a better reproducibility of results.

For the first time, using FLIM, the tests for TMZ sensitivity were performed on a heterogeneous group of samples, including primary and recurrent astrocytomas with various IDH status. This allowed us to assess the impact of previous treatment of patients on the metabolism of glioma cells and the in vitro response to TMZ treatment.

Lastly, we evaluated the correlations of all the fluorescence decay parameters (τ_2_, α_2_ and τ_m_) with the percentage of viable cells and the proliferative index to choose the most reliable marker of therapeutic response, which has not been carried out before.

On the one hand, NAD(P)H FLIM is a highly sensitive, label-free technique to follow heterogeneous drug response with single-cell resolution, which is advantageous compared to traditional methods that often report only on the average responsiveness of all cells in a population and require the use of dyes or immunofluorescence. On the other hand, FLIM also has some limitations. First, FLIM realized using a laser scanning microscope is overall technically complex and cost-prohibitive for widespread use in research labs and clinics. Second, there is a lack of standardized protocols for FLIM image acquisition and analysis, which is important for data reproducibility. Finally, the autofluorescent signal of NAD(P)H is generally much lower than that of any exogenous fluorophores, which imposes restrictions on the screening of drugs having their own fluorescence in the NAD(P)H spectral channel. Meanwhile, recent advances in optical technologies offer solutions for fluorescence lifetime measurements using more simple and cost-effective systems (such as confocal macro-FLIM or wide-field FLIM), which, in combination with the automatization of data analysis, opens the prospects for high-throughput drug testing in clinical settings.

One limitation of our study was the long time period (10–20 days) for cell culturing in our experiments required to obtain the sufficient amount of cells (about 15 × 10^5^ of cells) for the MTT-assay. However, the clinical application of this method will require a significantly lower amount of cells (0.5−1 × 10^5^ of cells) and, correspondingly, a shorter cultivation time period. We assume that in the clinical realization, a drug screening would be possible within 7–10 days after surgery.

We found that glioma cell response to TMZ depends on their metabolic status before treatment. The most pronounced changes in NAD(P)H fluorescence lifetime (increase in τ_m_), viability, and proliferation after chemotherapy were noticed in cell cultures with initially more oxidative metabolisms (higher τ_m_). For three of four glioma cultures with glycolytic metabolisms (lower τ_m_), a weak or no response to therapy was detected. The proposed mechanisms that mediate the drug resistance of glycolytic tumor cells include inhibition of apoptosis, induction of epithelial–mesenchymal transition, immunosuppression, induction of autophagy, inhibition of drug influx and increase of drug efflux, reducing cellular ROS level, elevated NADPH level from the pentose phosphate pathway, and a higher antioxidant capacity [22,23]. In general, upregulated glycolysis in gliomas often correlates with unfavorable prognosis [27]. These data indicate that the assessment of the metabolic states of glioma cells using FLIM may be a valuable prognostic tool.

Regarding the effects of TMZ on cell metabolism, we demonstrated that an increase in the NAD(P)H mean fluorescence lifetime tm has a strong linear correlation with a decrease in the percentage of viable cells and inhibited proliferation of cells after therapy, which ensure that the observed lifetime changes are due to response to treatment. Our results are consistent with the recent studies by Morelli et al. that show an increase in the fraction of bound NAD(P)H in TMZ-treated sensitive glioblastoma cell cultures [21]. Additionally, Wang et al. showed that TMZ-induced reprogramming of energy metabolism interferes with mitochondrial dynamics and increases oxidative phosphorylation levels without increasing ROS levels [28].

One limitation of our study is the short observation period of the patients, such that we could not compare the in vitro results with clinical outcomes for all the specimens tested. Since multiple factors determine the metabolic phenotype of a tumor, the assessment of FLIM data may be complicated by variable characteristics of the individual tumor, e.g., differentiation, mutations, grade, origin, and the presence and type of previous therapies. Further studies are needed to capture more material for validation purposes.

Nevertheless, the present study clearly demonstrates that FLIM of NAD(P)H enables drug screens directly on patient cells and may provide a platform for individual drug screening and drug development for glioblastoma patients. 

## 4. Materials and Methods

### 4.1. Patient’s Samples

All studies on tumor material were approved by the local ethics committee of PRMU (protocol #12 from 5 August 2022). Informed written consent was obtained from the patients prior to enrollment. The tumor specimens were obtained from the University Clinic at PRMU from 7 patients during tumor resectioning.

The samples of high-grade glioma (WHO grade 2–4) were transported in 50 mL Falcon tubes with DMEM (Dulbecco’s modified Eagle’s medium)/F12 medium with a twofold concentration of antibiotic–antimycotic (Gibco, Amarillo, TX, USA) on ice. Glioma cell cultures were obtained from each tumor sample and labeled as P1–P7.

The dataset consisted of 7 tumor specimens from 4 men and 3 women in the age group of 23–56 years. Each resected sample was labeled with information on grade, IDH-status (IDH-m or IDH-wt), primary or recurrent case and the survival, where appropriate. The IDH-status was identified by immunohistochemical staining (Supplementary Materials). Information about the samples is presented in Table 2.

### 4.2. Isolation of Primary Cells from Patient Samples

To obtain patient-derived glioma cell cultures, the standard protocol was used with minor optimizations [29]. The tumor specimen was washed 2–3 times in DMEM/F12 with 2% antibiotic–antimycotic to prevent contamination of the sample. Then, it was cut into 500 µm^3^ pieces and plated in 12-well plates (4–5 fragments per well) in RPMI-1640 medium with L-glutamine (Roswell Park Memorial Institute 1640 Medium), 10% fetal bovine serum (FBS), and 1% antibiotic–antimycotic in a CO_2_ incubator at 37 °C, 5% CO_2_, and 85% humidity. After 72 h of incubation, cells started to leave the explant, migrate radially, and initiate a “sun shape” formation. The medium was changed every 72 h and debris was removed. After 10–20 days, the glioma cells formed a monolayer with 80–90% confluency. The cells were removed from the plate by adding 300 mL of trypsin–EDTA (25%) to the well for 2–5 min and seeded on 25 cm^2^ culture flasks.

### 4.3. Cell Culturing

The patient-derived glioma cells were cultured in RPMI-1640 medium with L-glutamine with the addition of 10% fetal bovine serum (FBS) and 1% antibiotic–antimycotic (Gibco) on 25 cm^2^ culture flasks in a CO_2_ incubator at 37 °C, 5% CO_2_. Sub-cultivation was performed twice a week by adding 1 mL of trypsin–EDTA (25%) to the plate for 2–5 min. On the third passage, the cells were used in the experiments.

### 4.4. Immunofluorescence Staining

To evaluate the expression of GFAP, a standard marker of astroglial cells, the cells were grown in 30 mm Petri dishes to form a monolayer. Then, the cells were fixed with a solution of 4% paraformaldehyde for 10 min and permeabilized with a solution of 0.1% TritonX-100 for 5 min, incubated with primary antibodies to GFAP (Boster Biological Technology, Fremont, CA, USA) overnight at 4 °C, then incubated with secondary antibodies conjugated with the fluorescent dye AlexaFluor594 for 1 h at room temperature. The nuclei were contrasted with a fluorescent dye, DAPI. Imaging was performed using a Leica DMIL (Leica, Germany) wide-field fluorescence microscope equipped with a TX2 filter with 560/40 nm excitation and 645/75 nm emission for Alexa594 and a CFP filter with 436/20 nm excitation and 480/40 nm emission for DAPI.

### 4.5. Flow Cytometry

To evaluate the proliferative index, Ki67, the cells were removed from the plate, centrifuged at 200× *g* for 5 min, and resuspended in phosphate-buffered saline (PBS). The intracellular staining was performed using the Inside Stain Kit (Miltenyi Biotec, Bergisch Gladbach, Germany) according to the manufacturer’s protocol. The glioma cells were fixed, permeabilized, and stained with the antibody Ki67-Brilliant violet 425 (BioLegend, San Diego, CA, USA). The labeled cells were analyzed using a BD FACSAria III cell sorter (BD Biosciences, San Jose, CA, USA). The data were analyzed using FlowJo software (BD/Treestar, Ashland, OR, USA).

### 4.6. MTT Assay

Cell viability after treatment was measured using the MTT assay. MTT reagent, 3(4,5-dimethyl-2-thiasolyl)-2,5-diphenyl-2H-tetrasole bromide (Thermo Fisher Scientific, Waltham, MA, USA), was added to the growth medium in the final concentration of 0.5 mg/mL for 4 h. The absorbance was measured at 570 nm with a Synergy MX plate reader (BioTeck, Winooski, VT, USA). Cell viability was expressed as an optical density ratio between the treated and untreated cells. The cell viability was calculated as a percentage of untreated control cells. For each TMZ concentration, a MTT-assay was performed in 7–10 wells. The experiments were performed in triplicate. For each cell culture, the half-inhibitory concentration IC_50_ was calculated.

### 4.7. TMZ Drug Treatment

TMZ (Orion Corp./Shering, Finland) was used in this study. TMZ was dissolved in dimethyl sulfoxide (DMSO) to prepare a stock concentration of 175 mM and then diluted to the required concentrations with a complete cell culture medium. For drug treatment, glioma cells were seeded in 96-well plates (1.5 × 10^4^ per well) and cultured for 24 h.

For MTT-assay, TMZ was added to the cells at concentrations from 400 to 2000 µM. To perform the metabolic FLIM imaging, TMZ was added to the cells at the concentration of 1500 µM or 2500 µM. Equal volumes of DMSO were added to the control wells. The incubation time with TMZ was selected in the preliminary experiment (Supplementary Figure S3). In the present study, the cultures were exposed to TMZ for 72 h.

### 4.8. FLIM of NAD(P)H

For FLIM, the culture medium was changed to FluoroLite™ DMEM without phenol red (Gibco, USA) 30 min before the experiment.

FLIM of NAD(P)H was carried out using an LSM 880 laser scanning confocal microscope (Carl Zeiss, Jena, Germany) equipped with an FLIM module Simple Tau 152 TCSPC (Becker & Hickl GmbH, Berlin, Germany). A two-photon Ti:Sapphire femtosecond laser with 80 MHz and a pulse duration of 140 fs was used for excitation of NAD(P)H fluorescence at 750 nm. The fluorescence emission of NAD(P)H at 450–490 nm was selected by a combination of a 490 LP dichroic mirror and a ET475/50 bandpass filter (Chroma, US) and detected using a hybrid detector (HPM-100–40, Becker and Hickl GmbH, Germany). The average power used for the samples was about 6 mW. A C Plan-Apochromat 40×/1.3 NA Oil DIC objective was used for image acquisition. The field of view was 213 × 213 μm (512 × 512 pixels). The acquisition time of the images was 60 s.

The fluorescence decays of NAD(P)H were processed using the SPCImage 8.3 software (Becker and Hickl GmbH, Germany). On average, 5000–10,000 photons were collected per decay curve (binning factor 3) and the fitting was performed using a bi-exponential decay model by the weighted least square algorithm. The values of the short and long components of the lifetimes (τ_1_ and τ_2_) and their relative contributions (α_1_ and α_2_, α_1_ + α_2_ = 100%) were obtained, which correspond to the free and protein-bound forms of the NAD(P)H cofactor, respectively. The weighted average (mean) lifetime was calculated as τ_m_ = (α_1_ × τ_1_ + α_2_ × τ_2_)/(α_1_ + α_2_). In the images, the fluorescence lifetimes were analyzed in cell cytoplasm by manual selection of the maximal area of cytoplasm as the region of interest in each individual cell; the goodness of the fit χ^2^ was from 0.8 to 1.2. For each cell culture, FLIM images were acquired from 5 fields of view, a total of 25–40 cells. The detailed protocol for NAD(P)H FLIM can be found elsewhere [30].

### 4.9. Statistical Analysis

For statistical analysis and data presentation, a GraphPad Prism 8.0.1 (GraphPad, San Diego, CA, USA) was used. The normality of distribution was evaluated using the Shapiro–Wilk test (at *p* ≥ 0.05, the distribution was considered normal). Differences between groups were analyzed using Student’s *t*-test, and a *p* value ≤ 0.05 was considered statistically significant. The mean values (M) and standard error of the mean (SEM) were calculated.

## 5. Conclusions

The present study demonstrates that FLIM of NAD(P)H enables drug screens directly on patient cells and may provide a platform for individual drug screening and drug development for glioblastoma patients. Our results demonstrate that the more-responsive cell cultures displayed the longest mean fluorescence lifetime τm after TMZ treatment due to an increase in the protein-bound NAD(P)H fraction α_2_ associated with a shift to oxidative phosphorylation and the long lifetime component τ_2_ associated with a modified profile of NAD(P)H-binding proteins. The cell cultures that poorly responded to TMZ had generally shorter τm, i.e., were more glycolytic, and showed no or insignificant changes after treatment. The FLIM data correlate well with standard measurements of cellular drug response—cell viability, proliferation index, and clinical response in patients.

## Figures and Tables

**Table 1:** The characteristics of patient-derived glioma cell cultures.

	GFAP *	Ki67, %	Cell Polymorphism **	IC_50_ of TMZ, µM
P1	+	43 ± 2.1	+	1280 ± 13
P2	+	39 ± 2.6	+	476 ± 10
P3	+	56 ± 2.5	++	1266 ± 19
P4	+	59 ± 2.7	++	1680 ± 25
P5	+	53 ± 3.1	++	1757 ± 51
P6	+	45 ± 2.1	+	1098 ± 21
P7	+	70 ± 2.9	+	1673 ± 45

* + corresponds to GFAP-positive cultures; ** + or ++ corresponds to less or more pronounced cell polymorphism.

**Table 2:** The clinicopathological characteristics of patients.

Sample Code	Age	Sex	Grade	IDH-Status	Primary/Recurrent	Survival, Month
P1	39	M	4	Mutant	Recurrent	8
P2	56	M	4	NOS	Recurrent	25
P3	32	M	4	Mutant	Primary	>16
P4	52	F	4	Mutant	Recurrent	>14
P5	23	M	2	Mutant	Primary	>10
P6	46	F	4	Wild-type	Primary	>9
P7	40	F	3	Mutant	Primary	5

NOS (not otherwise specified)—the analysis of IDH-status was not performed.

## Data Availability

Data is contained within the article.

## Supplementary Materials

The following supporting information can be downloaded at: https://www.mdpi.com/article/10.3390/ph16060796/s1. Table S1. FLIM parameters of patient-derived glioma cultures with or without TMZ. Figure S1. The Pearson correlation between NAD(P)H τ_m_ in untreated and treated cell cultures and initial proliferation index. Scatter diagrams display the mean values for each glioma culture (dots) and the trend line. Figure S2. The Pearson correlations between NAD(P)H τ_2_ and α_2_ in untreated and treated cell cultures post-treatment viability and proliferation index. Scatter diagrams display the mean values for each glioma culture (dots) and the trend line. Figure S3. Quantification of τ_m_ in patient-derived glioma cultures without treatment and treated with TMZ for 24, 48 or 72 h at 1500 µM. Scatter dot plot displays the measurements for individual cells (dots) and the mean and SEM (horizontal lines). The labels below the horizontal axis indicate the individual patients. Figure S4. Quantification of τ_m_ in patient-derived glioma cultures without treatment and treated with TMZ for 72 h at 2500 µM. Scatter dot plot displays the measurements for individual cells (dots) and the mean and SEM (horizontal lines). The labels below the horizontal axis indicate the individual patients.
