# Supplementary material for: Measurement of Patient-Derived Glioblastoma Cell Response to Temozolomide Using Fluorescence Lifetime Imaging of NAD(P)H
Source: Pharmaceuticals (Basel). 2023 May 26;16(6):796. doi: 10.3390/ph16060796 (PMC10301219; doi:10.3390/ph16060796)
Supplement: Supplementary file 1 [file pharmaceuticals-16-00796-s001.zip › pharmaceuticals-2341221-supplementary.pdf]

Table S1. FLIM parameters of patient-derived glioma cultures with or without TMZ

Sample code	Treatment	τ_m, ns	τ_1, ns	τ_2, ns	α_1, %	α_2, %
P7	Non-treated	0.62 ± 0.01	0.25 ± 0.003	1.90 ± 0.01	77.4 ± 0.3	22.6 ± 0.3
	TMZ-treated	0.64 ± 0.01	0.26 ± 0.01	1.94 ± 0.02	77.5 ± 0.2	22.5 ± 0.2
P5	Non-treated	0.65 ± 0.01	0.26 ± 0.004	1.994 ± 0.02	77.17± 0.3	22.83 ± 0.3
	TMZ-treated	0.71 ± 0.004<br>$p < 0.0001$	0.30 ± 0.003<br>$p < 0.0001$	2.15 ± 0.01<br>$p < 0.0001$	77.55 ± 0.2	22.45±0.2
P4	Non-treated	0.67 ± 0.01	0.27 ± 0.01	2.09 ± 0.03	77.88 ± 0.2	22.03 ± 0.2
	TMZ-treated	0.71 ± 0.01<br>$p = 0.005$	0.29 ± 0.004<br>$p = 0.0025$	2.12 ± 0.02	77.2 ± 0.2	22.8 ± 0.2
P3	Non-treated	0.70 ± 0.01	0.27 ± 0.01	2.05 ± 0.02	75.9 ± 0.2	24.1 ± 0.2
	TMZ-treated	0.74 ± 0.01<br>$p = 0.0237$	0.31 ± 0.01<br>$p < 0.0001$	2.25 ± 0.03<br>$p < 0.0001$	77.7 ± 0.4<br>$p = 0.0002$	22.3 ± 0.4<br>$p = 0.0002$
P1	Non-treated	0.78 ± 0.01	0.33 ± 0.01	2.3 ± 0.03	76.8 ± 0.3	23.2 ± 0.3
	TMZ-treated	0.88 ± 0.02<br>$p < 0.0001$	0.33 ± 0.01	2.4 ± 0.04	72.7 ± 0.5<br>$p < 0.0001$	27.3 ± 0.5<br>$p < 0.0001$
P6	Non-treated	0.72 ± 0.01	0.28 ± 0.004	2.13 ± 0.02	76.4 ± 0.2	23.6 ± 0.2
	TMZ-treated	0.88 ± 0.01<br>$p < 0.0001$	0.3 ± 0.004<br>$p = 0.0040$	2.36 ± 0.02<br>$p < 0.0001$	71.6 ± 0.3<br>$p < 0.0001$	28.4 ± 0.3<br>$p < 0.0001$
P2	Non-treated	0.88 ± 0.01	0.37 ± 0.01	2.37 ± 0.03	74.1 ± 0.3	25.9 ± 0.3
	TMZ-treated	1.03 ± 0.01<br>$p < 0.0001$	0.45 ± 0.01<br>$p < 0.0001$	2.55 ± 0.03<br>$p < 0.0001$	72.1 ± 0.3<br>$p < 0.0001$	27.9 ± 0.3<br>$p < 0.0001$

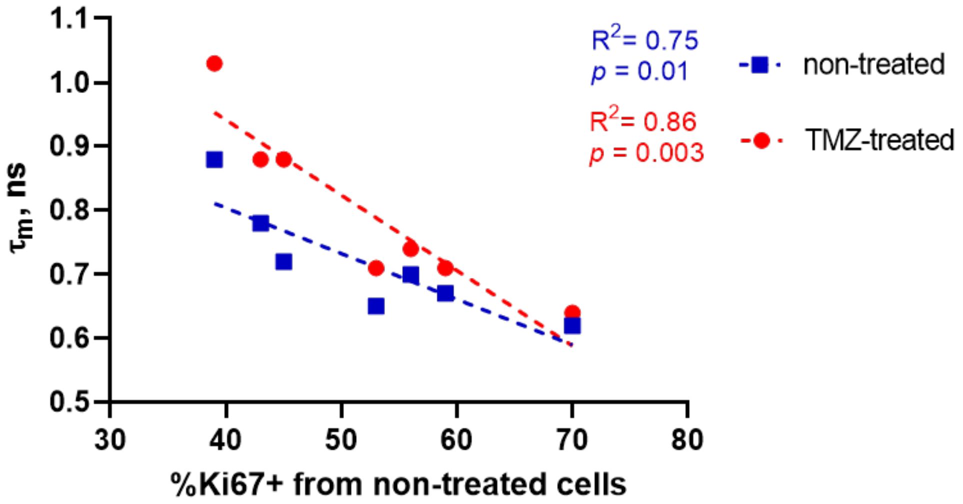

Figure S1. The Pearson correlation between NAD(P)H τ_m in untreated and treated cell cultures and initial proliferation index. Scatter diagrams display the mean values for each glioma culture (dots) and the trend line.

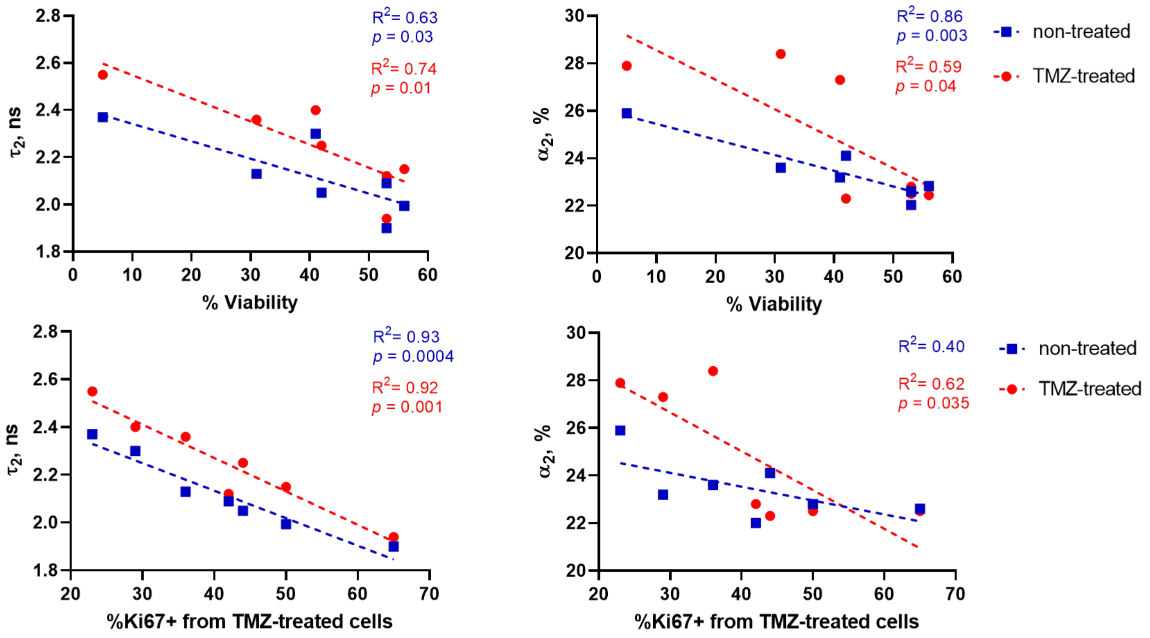

Figure S2. The Pearson correlations between NAD(P)H τ_2 and α_2 in untreated and treated cell cultures post-treatment viability and proliferation index. Scatter diagrams display the mean values for each glioma culture (dots) and the trend line.

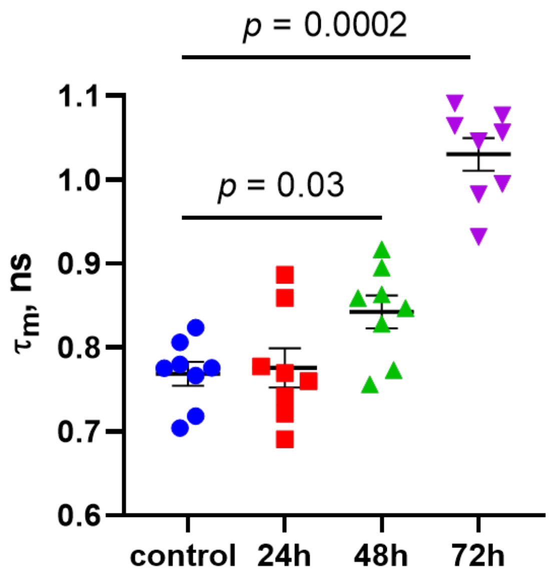

Figure S3. Quantification of τ_m in patient-derived glioma cultures without treatment and treated with TMZ for 24, 48 or 72 h at 1500 µM. Scatter dot plot displays the measurements for individual cells (dots) and the mean and SEM (horizontal lines). The labels below the horizontal axis indicate the individual patients.

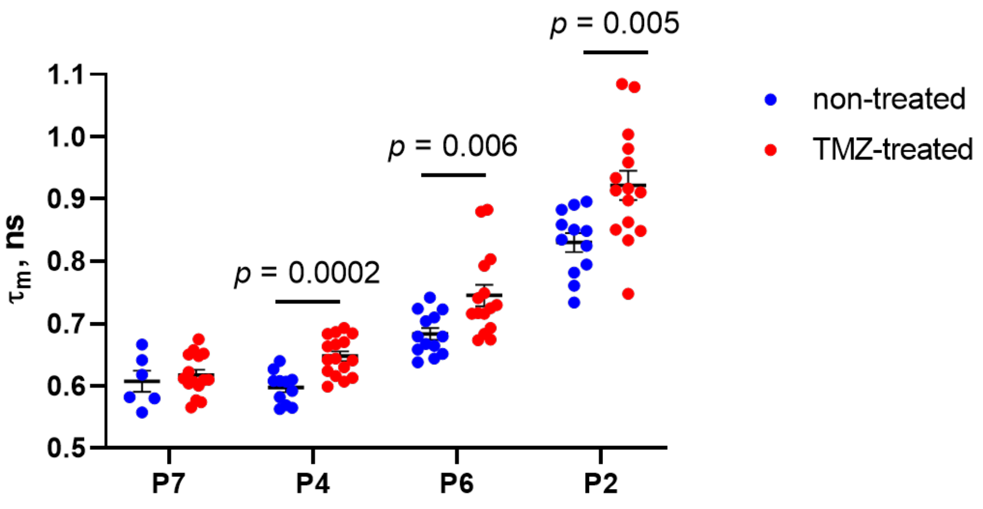

Figure S4. Quantification of τ_m in patient-derived glioma cultures without treatment and treated with TMZ for 72 h at 2500 µM. Scatter dot plot displays the measurements for individual cells (dots) and the mean and SEM (horizontal lines). The labels below the horizontal axis indicate the individual patients.

Supplementary Materials and Methods

Immunohistochemistry

Immunohistochemical staining was performed as follows: 2-µm-thick sections were prepared from formalin-fixed paraffin-embedded TMA tissue blocks and were dried in a 60 °C oven overnight. The sections were placed in a BOND-MAX Automated Immunohistochemistry Vision Biosystem (Leica Microsystems GmbH, Wetzlar, Germany) according to the following protocol. First, tissues were deparaffinized and pre-treated with the Epitope Retrieval Solution 2 (EDTA-buffer pH8.8) at

98 °C for 20 min. After washing steps, peroxidase blocking was carried out for 10 min using the Bond Polymer Refine Detection Kit DC9800 (Leica Microsystems GmbH). Tissues were again washed and then incubated with the primary antibody for 30 min. Anti-IDH1 R132H / DIA-H09 Mouse monoclonal anti-brain tumor marker Clone H09 (Dianova, Hamburg, Germany) was used. Subsequently, tissues were incubated with polymer for 10 min and developed with DAB-Chromogen for 10 min. A cytoplasmic and perinuclear immunoreaction for IDH 1 were scored as positive.
